# Cigarette smoke represses the innate immune response to asbestos

**DOI:** 10.14814/phy2.12652

**Published:** 2015-12-10

**Authors:** Gilbert F. Morris, Svitlana Danchuk, Yu Wang, Beibei Xu, Roy J. Rando, Arnold R. Brody, Bin Shan, Deborah E. Sullivan

**Affiliations:** ^1^ Departments of Pathology and Laboratory MedicineProgram in Lung BiologyNew OrleansLouisiana; ^2^ Microbiology and ImmunologyProgram in Lung BiologyNew OrleansLouisiana; ^3^ Global Environmental Health SciencesTulane University Health Sciences CenterProgram in Lung BiologyNew OrleansLouisiana; ^4^ College of Medical SciencesWashington State University SpokaneProgram in Lung BiologySpokaneWashington

**Keywords:** Asbestos, cigarette smoke, IL‐18, IL‐1*β*, NLRP3 inflammasome

## Abstract

Both cigarette smoke (CS) and asbestos cause lung inflammation and lung cancer, and at high asbestos exposure levels, populations exposed to both of these carcinogens display a synergistic increase in the development of lung cancer. The mechanisms through which these two toxic agents interact to promote lung tumorigenesis are poorly understood. Here, we begin to dissect the inflammatory signals induced by asbestos in combination with CS using a rodent inhalation model and in vitro cell culture. Wild‐type C57BL/6 mice were exposed to room air as a control, CS, and/or asbestos (4 days per week to CS and 1 day per week to asbestos for 5 weeks). Bronchoalveolar lavage (BAL) fluid was collected following exposure and analyzed for inflammatory mediators. Asbestos‐exposed mice displayed an increased innate immune response consistent with NLRP3 inflammasome activation. Compared to mice exposed only to asbestos, animals coexposed to CS + asbestos displayed attenuated levels of innate immune mediators and altered inflammatory cell recruitment. Histopathological changes in CS + asbestos‐exposed mice correlated with attenuated fibroproliferative lesion development relative to their counterparts exposed only to asbestos. In vitro experiments using a human monocyte cell line (THP‐1 cells) supported the in vivo results in that coexposure to cigarette smoke extract repressed NLRP3 inflammasome markers in cells treated with asbestos. These observations indicate that CS represses central components of the innate immune response to inhaled asbestos.

## Introduction

Chronic inflammation underlies the pathogenesis of many lung diseases including lung cancer (Gomperts et al. 2011). In accord, two established lung carcinogens, cigarette smoke (CS) and asbestos, cause inflammation in the lung (Donaldson 1996; Malkinson 2005; Takahashi et al. 2010). Combined inhalation exposures to CS and asbestos elicit a synergistic to more than additive increase in human lung cancers depending on the level(s) of exposure (Erren et al. 1999; Henderson et al. 2004; Markowitz et al. 2013; Markowitz 2015). However, how repeated, sequential inhalation exposures to CS and asbestos affect lung inflammation has not been described.

Inhaled asbestos and silica initiate inflammation through activation of the NOD‐like receptor family, pyrin domain containing 3 (NLRP3) inflammasome (Cassel et al. 2008; Dostert et al. 2008). Production of reactive oxygen species (ROS) and consequent release of the thioredoxin‐interacting protein induces the assembly of the inflammasome complex (Zhou et al. 2010; Thompson et al. 2014). Upon activation, NLRP3 and the adapter apoptosis‐associated speck‐like protein containing a caspase recruitment domain (ASC) form the multisubunit inflammasome that facilitates autocatalytic cleavage of inactive procaspase‐1 to its active form, which cleaves pro‐IL‐1*β* and pro‐IL‐18 to their mature secreted forms (Schroder and Tschopp 2010). NLRP3 null mice have defects in IL‐1*β* secretion and immune cell recruitment following asbestos exposure (Chow et al. 2012). In addition, asbestos instillation potentiates release of high mobility group box 1 protein (HMGB1) (Yang et al. 2010), which serves as a danger‐associated molecular pattern (DAMP) or alarmin, that initiates proinflammatory signaling through binding the toll‐like receptors (TLR2 and TLR4) and the receptor for advanced glycation end‐products (RAGE) (Sims et al. 2010). HMGB1 can also play a regenerative role by stimulating cell proliferation (Limana et al. 2005). The expression of these factors supports the concept that asbestos exposure induces an innate immune response in humans and experimental animals.

Inhalation of CS induces neutrophilia in the lung (Cosio et al. 2009) that varies in severity in different strains of mice (Pouwels et al. 2015). Reduction in lung eosinophils occurs after brief CS exposures of rats (Jeffery and Reid 1981) and mice (Melgert et al. 2004). This correlates with an increase in interleukin‐8 and a decrease in eosinophils in the blood that precedes an increase in neutrophils and lymphocytes in the sputum during the acute response to CS in humans (van der Vaart et al. 2005). The inflammatory response to CS requires IL‐1RI (Doz et al. 2008; Churg et al. 2009; Pauwels et al. 2011) and appears to be affected by duration of exposure and reliant upon IL‐1*α* (Pauwels et al. 2011; Eltom et al. 2014). Lung injury due to oxidative stress likely accounts for the inflammation that develops in the lungs of CS‐exposed mice (Yang et al. 2006; Lagente et al. 2008; Nemmar et al. 2012). CS exposure increases intracellular levels of HMGB1 in the lungs of mice (Bezerra et al. 2011), but elicits only a modest transient release of this alarmin (Heijink et al. 2015). Similarly, CS enhances epithelial expression, but not release, of IL‐33, an alarmin that is released upon viral infection (Kearley et al. 2015).

Epidemiologic studies indicate that interactions between cigarette smoke and asbestos contribute to lung carcinogenesis (Erren et al. 1999) for all histologic subtypes (Vainio and Boffetta 1994), but the nature of those interactions remains largely undefined. Cigarette smoke impairs asbestos clearance in the lung (Churg et al. 1987), and both of these carcinogens cause oxidative stress with associated DNA damage, which induces cell signaling for survival and proliferation (Mossman et al. 2006). The combined effects of cigarette smoke and asbestos upon lung inflammation remain to be determined. Thus, the goal of the work described here is to characterize the inflammatory response in the lungs of mice exposed to the combination of these two carcinogens and to understand the mechanisms that can lead to enhanced tumor formation. We postulate that inhaled asbestos fibers activate components of the innate immune system and that cigarette smoke represses this response, resulting in reduced fiber clearance and chronic inflammation, thus explaining in part the increased risk of developing lung cancer.

## Materials and Methods

### Animal exposures

The institutional animal care and use committee approved all procedures with animals. Wild‐type C57BL/6 (B6) mice 8–10 weeks of age were obtained from the National Cancer Institute‐Frederick. In one experiment, wild‐type B6 mice were derived from breeding pairs in the Tulane vivarium. All mice were housed in an Association for Assessment and Accreditation of Laboratory Animal Care accredited facility according to National Institutes of Health guidelines. Mice were maintained on a 12‐h light/12‐h dark schedule and given food and water ad libitum.

Mice were exposed “nose only” for 5 h once per week for 5 weeks to an aerosol of chrysotile asbestos at an average density of 17.1–21.2 mg/m^3^. Control animals were exposed to filtered room air. The exposure atmospheres were produced with a Timbrel generator loaded with NIEHS medium grade chrysotile asbestos (Pinkerton et al. 1983). The output from the generator passed through a vertical elutriator, which removed particles with aerodynamic diameters greater than about 10 *μ*m, prior to entering the nose‐only exposure chamber (Hammad et al. 1982). Optical microscopic size distribution analysis of aerosolized fiber samples collected from the exposure chamber yielded geometric mean fiber diameter of 0.54 ± 0.06 *μ*m with a geometric standard deviation of 1.32 ± 0.06, geometric mean fiber length of 7.50 ± 0.75 *μ*m with geometric standard deviation of 2.45 ± 0.26, and geometric mean fiber aerodynamic diameter (calculated according to Reist [Reist 1993]) of 1.98 ± 0.27 *μ*m with geometric standard deviation of 1.44 ± 0.08.

CS exposures were whole body for 5 h per day, 4 days per week for 5 weeks to environmental cigarette smoke generated from KY3R4F reference cigarettes (9.4 mg tar, 0.73 mg nicotine per cigarette) purchased from the Tobacco Research Institute, University of Kentucky in a model TE‐10 smoking machine (Teague Enterprises, Davis, CA) as described previously (Izzotti et al. 2004). Briefly, environmental CS is 90% side‐stream smoke and 10% mainstream smoke generated by simultaneously burning three cigarettes that produce approximately 150 mg/m^3^ total suspended particles (TSP). The cigarettes were puffed for 2 sec at a flow rate of 1.05 L/min to provide a standard puff of 35 cm^3^. CS exposures were monitored every 1.5 h by sampling the total particulate matter per cubic meter of air in the exposure chamber.

### Bronchoalveolar lavage and analyses

After administration of anesthesia, blood was collected by heart puncture and mice were euthanized by exsanguination upon clipping the renal artery. Bronchoalveolar lavage (BAL) of mice was performed with 0.8 mL × 5 lavage buffer (phosphate buffered saline, 0.4 mmol/L ethylene diamine tetra acetic acid) as described previously (Guenther et al. 2010). The recovered BAL fluid from the first lavage was aliquoted before freezing at −70°C for cytokine profiling. Total BAL protein was determined by bicinchoninic acid (BCA) assay (Pierce). The total cells from the first and subsequent BALs were recovered by centrifugation at 1500 *g* for 5 min at 4°C and pooled. The live cells were identified by trypan blue exclusion and quantified by counting with a hemocytometer. Cytospin slides were prepared from the total recovered cells and blinded differential cell counts were performed after Hema 3 (Fisher Scientific, Waltham, MA) staining of each slide.

Levels of lactate dehydrogenase activity in the BAL fluid were determined using the BioVision LDH cytotoxicity assay kit II. HMGB1 levels in the BAL fluid of the exposed mice were determined by ELISA (IBL International, Hamburg, Germany). Caspase‐1 activity in the BAL fluid was measured by colorimetric assay (BioVision, Zurich, Switzerland). Cytokine profiling of BAL samples was performed as described previously (Guenther et al. 2010) on a Luminex Bio‐Plex 200 system using the Bio‐Plex Mouse Cytokine 23‐Plex Panel (Bio‐Rad, Hercules, CA) or the Milliplex mouse 22 cytokine/chemokine and 6‐plex panels (Millipore, Billerica, MA). Cytokine standards were reconstituted and diluted in lavage buffer. Bovine serum albumin (0.5%) was added to samples and standards as carrier protein except for the Milliplex cytokine/chemokine panels. All subsequent steps were performed according to the suppliers’ instructions. Levels of IL‐18 in the BAL fluid were determined by ELISA according to the supplier's instructions (Medical and Biological Laboratories, Naka‐ku Nagoya, Japan).

### Histopathology

After BAL, the right mainstem bronchus was ligated, the right lung was removed and quickly frozen in liquid nitrogen. The left lung was perfused via a catheter inserted into the trachea with 10% neutral buffered formalin at 30 cm pressure for 20 min. The trachea was ligated and the catheter was removed before removal of the left lung, which was then fixed overnight in 10% formalin before transfer to phosphate‐buffered saline (PBS) the next day. The fixed left lung was embedded in paraffin and 5 *μ*m sections were stained with hematoxylin and eosin (H&E) to reveal the anatomic details. The H&E stained slides were scanned at 400× magnification with an Aperio slide scanner. Using scanner software, the perimeter and area of first alveolar duct bifurcations delineated by the first alveolar wall to transect the axis were measured in a blinded manner. Lesion size was determined by the ratio of the area (measured in *μ*m^2^) to the perimeter (measured in *μ*m).

### Cell culture

The human monocyte cell line THP‐1 was obtained from American Type Culture Collection and cultured in RPMI supplemented with 10% heat‐inactivated fetal bovine serum, 5 μmol/L 2‐mercaptoethanol (complete medium). The monocyte‐like cells in suspension were differentiated into more macrophage‐like adherent cells by overnight treatment with 5 ng/mL phorbol 12‐myristate 13‐acetate (PMA, Enzo life sciences). The following day the adherent cells were washed twice with PBS and exposed to cigarette smoke extract (CSE) and/or asbestos in fresh complete medium. For asbestos fiber exposure, chrysotile asbestos was diluted in PBS and then sheared by 10 sequential passages through increasing gauge needles 18–25. The resulting slurry was bath sonicated for 2 h prior to addition to cell culture. The cigarette smoke extract (CSE) was prepared by bubbling smoke from a KY3R4F research cigarette in 25 mL of complete medium. The resulting CSE media was labeled as 100% and then diluted to the desired concentration (20% unless otherwise stated) in complete medium. IL‐1*β* in THP‐1 cell supernatants was measured by ELISA according to the manufacturer's instructions (Biolegend, San Diego, CA). For immunoblotting studies, the THP‐1 cells growing in complete medium were differentiated by the addition of 200 ng/mL PMA for 3 h, before washing with PBS. The cells were seeded at 1 × 10^6^ cells per mL in opti‐MEM (Life Technologies, Carlsbad, CA) and allowed to adhere overnight. After 6 h with 20 *μ*g/cm^2^ asbestos and/or 20% CSE treatment in opti‐MEM, the cell culture supernatant was removed, centrifuged at 10,000 *g* for 10 min and frozen at −80°C. IL‐18 was measured in the cell culture supernatants by ELISA according to the supplier's specifications (eBioscience, San Diego, CA). The cells were lysed in SDS lysis buffer (62.5 mmol/L Tris‐HCl, pH6.8, 2% SDS, 10% glycerol, 50 mmol/L DTT). The supernatant (0.75 mL) was thawed, mixed by vortexing with 0.75 mL methanol plus 0.1875 mL chloroform, and centrifuged for 5 min at 16,000 *g*. The upper phase was removed without disturbing the interface and the protein was precipitated by adding 0.75 mL methanol and vortexing before collection by centrifugation at 16,000 *g* for 5 min. The protein pellet was briefly dried under vacuum before dissolving in 80 *μ*L SDS lysis buffer followed by boiling for 10 min for immunoblot analyses. Equal amounts of protein were separated by SDS‐PAGE (4–12% Bis‐Tris gradient precast NuPAGE gel, Invitrogen, Carlsbad, CA) and transferred to nitrocellulose. The blots were blocked with 5% BSA and incubated overnight at 4°C with primary antibodies (rabbit anticaspase‐1, EMD Millipore; goat anticleaved caspase‐1, Santa Cruz; mouse monoclonal anti‐IL‐*β*, Cell Signaling) diluted in blocking solution at the recommended concentrations. Following three washes with Tris‐buffered saline (TBS), blots were incubated with appropriate horseradish peroxidase‐conjugated secondary antibodies (Life Technologies) diluted in 5% BSA at 1:10,000. After washing in TBS, proteins were detected using SuperSignal chemiluminescent substrate (Pierce Biotechnology, Rockford, IL) according to the manufacturer's instructions. Membranes were then stripped and reprobed using a mouse monoclonal antibody to *β* actin (Abcam, Cambridge, UK) to correct for differences in protein loading. Densitometric analysis of western blot images was performed using UN‐SCAN‐IT^™^ software (Silk Scientific, Inc., Orem, UT).

### Statistical analyses

In some instances, values were normalized to the asbestos‐exposed group. Unless indicated otherwise, the data are reported as mean ± SEM. Significance between groups and post hoc pair comparisons were determined by Kruskal–Wallis one‐way ANOVA for animal exposures and ordinary one‐way ANOVA for cell culture experiments using Prism 6 software for statistical analysis.

## Results

**Figure 1:**
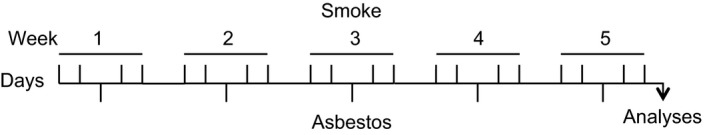
Scheme for inhalation exposure of mice to cigarette smoke and/or asbestos. B6 mice were exposed for 5 h per day/5 days per week/5 weeks.

**Figure 2:**
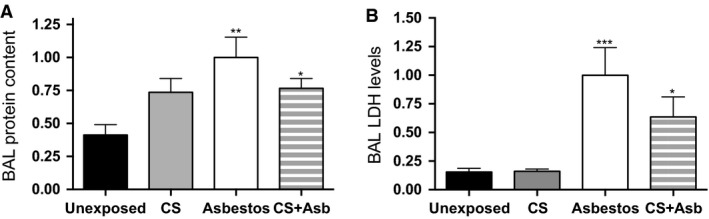
Interaction between inhaled cigarette smoke and asbestos in inflammatory cell recruitment and lung injury. B6 mice were exposed to air, CS, asbestos, or CS + asbestos according to the scheme depicted in Figure 1. BAL fluid was prepared from mice euthanized on the day after the last smoke exposure (day 3 after the last asbestos exposure). The graphs display mean values ± SEM for mice exposed to air (black bars; *n* = 9), CS (gray bars; *n* = 5), asbestos (white bars; *n* = 10), and CS + asbestos (gray‐white stripes, *n* = 12) from two separate exposures normalized to values in the asbestos‐exposed group. (A) BAL protein content. The protein concentration in the BAL fluid as determined by BCA protein assay for the indicated exposure is shown (*P* = 0.0054, Kruskal–Wallis ANOVA; **P < 0.01, unexposed vs. asbestos; *P < 0.05, unexposed vs. CS + asbestos). (B) BAL lactate dehydrogenase (LDH) activity. The BAL fluid from exposed mice was assayed for LDH by colorimetric assay in a microtiter plate format. The graph shows relative LDH activity in the BAL fluid from mice in the indicated exposure group normalized to the asbestos‐exposed group (*P* = 0.0002, Kruskal–Wallis ANOVA; ***P < 0.001, unexposed vs. asbestos; *P < 0.05, unexposed vs. CS + asbestos).

Since CS and asbestos exposure can synergize in a dose‐dependent manner to induce lung carcinogenesis in humans (Erren et al. 1999; Henderson et al. 2004; Markowitz et al. 2013), we anticipated that inhalation of CS by mice would exacerbate asbestos‐induced lung injury and inflammation. To test this postulate, we exposed wild‐type C57BL/6 (B6) mice 5 h per day to air or to CS (~150 mg/m^3^ TSP) alone 4 days per week, and to asbestos (~20 mg/m^3^) alone 1 day per week for 5 weeks. This level of whole body CS exposure, or less (90 mg/m^3^), for 5 h/day, 5 days/week for 6 months is sufficient to induce emphysema in mice (Sussan et al. 2009; Eppert et al. 2013). The fourth group of mice received both CS plus asbestos by sequentially exposing them to CS and asbestos in a 2:1:2 (CS:asbestos:CS) daily format 5 days per week for 5 weeks as well (Fig. 1). A preliminary study with mice exposed only to asbestos followed by daily analysis of the BAL fluid of the exposed animals showed that induction of IL‐1*β* was observed on day 3 postexposure (data not shown). Thus, in the combined exposures, BAL was performed 1 day after the last CS exposure, which corresponded to day 3 after the last asbestos exposure. Exposure to CS alone had no effect on markers of injury, BAL total protein, or lactate dehydrogenase (LDH) (Fig. 2A and B). Total protein and LDH increased with exposure to asbestos and CS coexposure had a small, but insignificant effect upon these markers of lung injury (Fig. 2A and B).

### Cigarette smoke alters lung inflammation induced by inhaled asbestos

**Figure 3:**
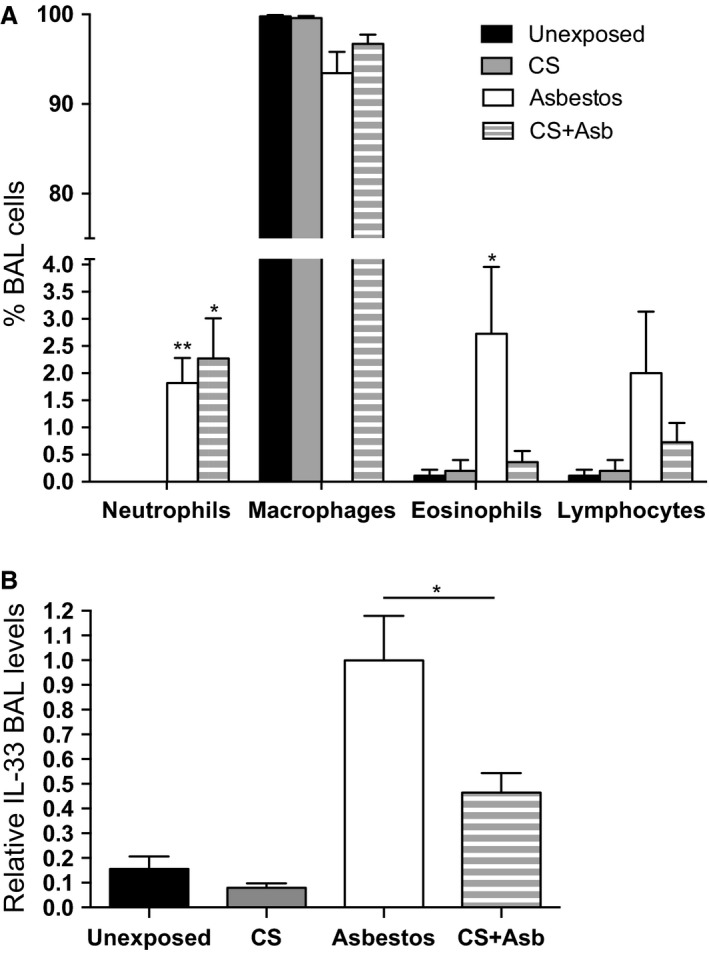
Inhalation of cigarette smoke alters inflammation and alarmin release in asbestos‐exposed mice. (A) Differential cell counts of BAL cells. Cytospin samples of the cells recovered by BAL (see Fig. 2) were stained with Hema 3. Cells were visualized by microscopy and 100 cells were counted from each blinded sample. The graph shows the percentage of the indicated cell type (mean ± SEM) in the cells recovered from the BAL fluid from mice exposed to air, CS, asbestos, and CS + asbestos. The percentage of neutrophils in the BAL increased relative to unexposed control mice (*P* = 0.001, Kruskal–Wallis ANOVA) in mice exposed to asbestos (**P < 0.01) and CS + asbestos (*P < 0.05). Only exposure to asbestos increased (*P < 0.05) the number of BAL eosinophils (*P* = 0.001, Kruskal–Wallis ANOVA). (B) IL‐33 levels in the BAL fluid from exposed mice. BAL fluid from mice described in Figure 2 was assessed for IL‐33 by multiplex ELISA (Millipore). For the purposes of comparison, the levels of IL‐33 in asbestos‐exposed mice were normalized to 1.0 (*P* < 0.0001, Kruskal–Wallis ANOVA; *P < 0.05, asbestos vs. CS + asbestos).

Differential cell staining of the inflammatory cells in the BAL fluid showed that air‐ or CS‐exposed lungs contained primarily macrophages (Fig. 3A). The C57BL/6 strain used in our experiments is known to have lower lung neutrophil numbers after cigarette exposure than some other strains (Morris et al. 2008). Inhalation exposure to asbestos modestly (~2–3%) increased lung levels of neutrophils and eosinophils. The addition of CS to the asbestos exposure had no effect upon lung neutrophils, but repressed the asbestos‐associated increase in eosinophils (Fig. 3A).

Cell injury can cause release of alarmins or *d*amage *a*ssociated *m*olecular *p*atterns (DAMPS), such as high mobility group 1 (HMGB1) and interleukin‐33 (IL‐33), which display similarities in nuclear localization and ability to activate the immune system (Arshad et al. 2012). Levels of HMGB1 increased, but not significantly, in asbestos‐exposed mice relative to their unexposed counterparts (data not shown). Mice exposed to CS + asbestos displayed levels of HMGB1 that were ~30% lower than in mice exposed to asbestos alone, but the group differences did not quite reach significance (*P* = 0.058, Kruskal–Wallis ANOVA). More dramatic differences were observed with IL‐33, which was increased >sixfold in asbestos‐exposed mice relative to the unexposed controls. Exposure of mice to CS + asbestos significantly reduced levels of IL‐33 in the BAL fluid by approximately 50% (Fig. 3B). These observations are consistent with our postulate that inhaled CS dampens the innate immune response to inhaled asbestos.

### Cigarette smoke represses activation of the NLRP 3 inflammasome in vivo

**Figure 4:**
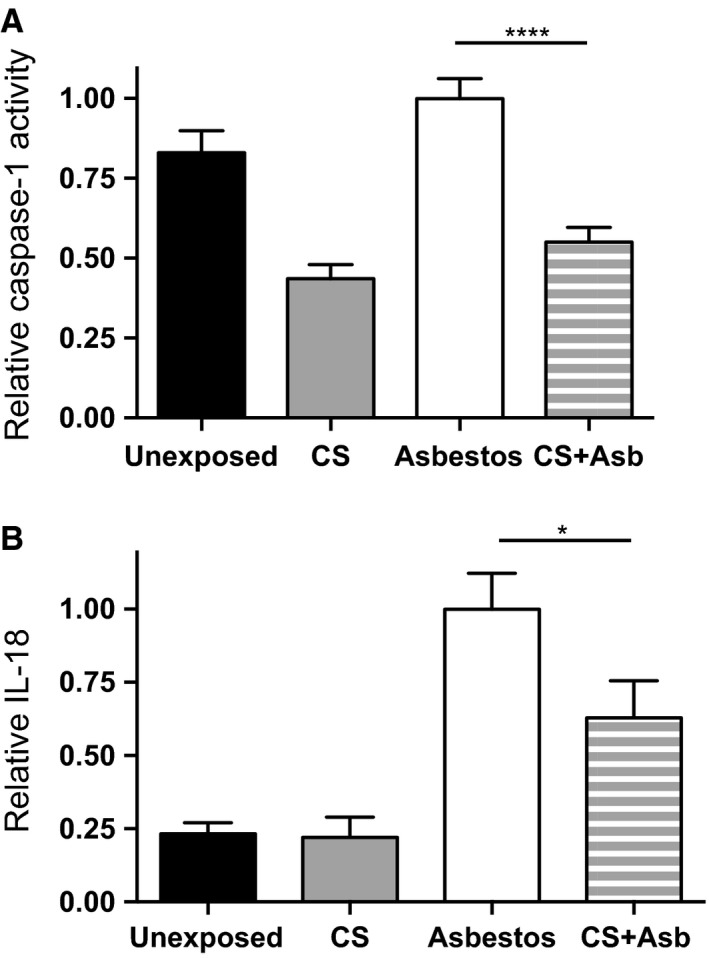
Inhalation of cigarette smoke represses activation of the NLRP3 inflammasome in asbestos‐exposed mice. In three separate exposures, B6 mice were exposed to air, CS, asbestos, or CS + asbestos according to the scheme depicted in Figure 1. BAL fluid was prepared from the mice on the day after the last smoke exposure (day 3 after the last asbestos exposure). The graphs display mean values ± SEM for mice exposed to air (black bars; *n* = 14), CS (gray bars; *n* = 11), asbestos (white bars; *n* = 17), and CS + asbestos (gray‐white stripes, *n* = 18). (A) Active caspase‐1. Values for active caspase‐1 in the BAL fluid were normalized to those of asbestos‐exposed mice in each group. Active caspase‐1 levels in the BAL fluid from asbestos‐exposed mice were approximately 1.8‐fold higher (****P < 0.0001) in asbestos‐exposed mice than in mice exposed to CS + asbestos (*P* < 0.0001, Kruskal–Wallis ANOVA). (B) IL‐18 levels. Same as part A except IL‐18 levels in the BAL fluid of exposed mice were determined by ELISA and values were normalized to asbestos‐exposed mice. Levels of IL‐18 were fourfold higher in asbestos‐exposed mice than in unexposed controls (*P* < 0.0001, Kruskal–Wallis ANOVA). Mice exposed to CS + asbestos displayed significantly lower BAL levels of IL‐18 relative to asbestos‐exposed mice (*P < 0.05).

In addition to cell injury‐mediated immune activation, inhaled asbestos promotes lung inflammation by activating the NLRP3 inflammasome (Cassel et al. 2008; Dostert et al. 2008). The activated NLRP3 inflammasome facilitates activation of caspase‐1 via autocatalytic cleavage of procaspase‐1. Active caspase‐1 subsequently cleaves immature proforms of the inflammatory cytokines IL‐1*β* and IL‐18 to mature active forms prior to secretion (Sutterwala et al. 2014). To assess the effect of CS on asbestos‐mediated secretion of inflammasome‐dependent cytokines, we measured levels of caspase‐1 in the BAL fluid from mice in the four exposure groups described earlier. Inhalation exposure to asbestos increased levels of active caspase‐1 and coexposure to CS significantly reduced that induction (Fig. 4A). Although statistically insignificant (*P* = 0.056, Kruskal–Wallis ANOVA), but consistent with the caspase‐1 findings, slightly elevated levels of IL‐1*β* in asbestos‐exposed mice were repressed ~30% upon CS + asbestos coexposure (data not shown). A more pronounced asbestos‐induced increase in lung IL‐18 levels was significantly reduced by CS + asbestos coexposure (Fig. 4B).

### Histopathological changes associated with cigarette smoke + asbestos exposure

**Figure 5:**
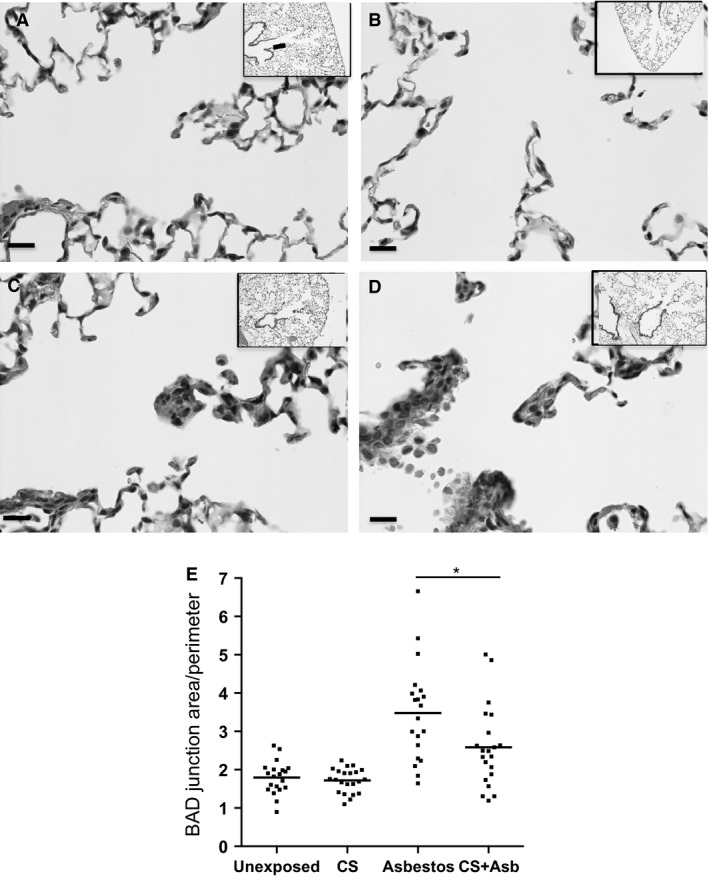
Histopathological alterations associated with cigarette smoke + asbestos coexposure. Formalin‐fixed paraffin embedded lung tissue sections from mice in the four exposure groups were H&E stained and photographed at 400× magnification (bar in each panel equals 10 microns L). The boxed areas represent the bronchiolar–alveolar duct (BAD) junctions for (A) air‐, (B) CS‐, (C) asbestos‐, and (D) CS + asbestos‐exposed mice. (E) Measurements of the BAD junction area (*μ*m^2^) and perimeter (*μ*m) were determined by image analyses of H&E stained sections with an Aperio slide scanner. The graph shows the area/perimeter ratio for each BAD junction (average of 7 BAD junctions per mouse × 3 mice per exposure group) for the indicated exposure. Exposure of mice to asbestos produced lesions at the BAD junction that were approximately 40% larger (*P < 0.05) than BAD junction lesions in CS + asbestos‐exposed mice (*P* < 0.0001, Kruskal–Wallis ANOVA).

Inhaled asbestos fibers small enough to pass through the conducting airways deposit at the ends of the terminal bronchioles at the bronchiolar–alveolar duct (BAD) junctions to produce characteristic lesions at these sites (Brody and Roe 1983; Chang et al. 1988). The BAD junctions of unexposed mice (Fig. 5A) could not be distinguished from those in mice exposed only to CS (Fig. 5B), whereas inhalation exposure to asbestos (Fig. 5C) or CS + asbestos (Fig. 5D) produced the expected fibroproliferative lesions at these sites. We showed previously in the rat inhalation model that asbestos alone induces significantly enhanced proliferation of airway epithelial and mesenchymal lining cells as well as endothelial and smooth muscle cells of small vessels (McGavran and Brody 1989; McGavran et al. 1990). In this study, area/perimeter measurements of the BAD junctions were analyzed to assess lesion development. Results showed that coexposure to CS significantly attenuated development of asbestos lesions by ~50% (Fig. 5E).

### Cigarette smoke represses activation of the NLRP 3 inflammasome in vitro

**Figure 6:**
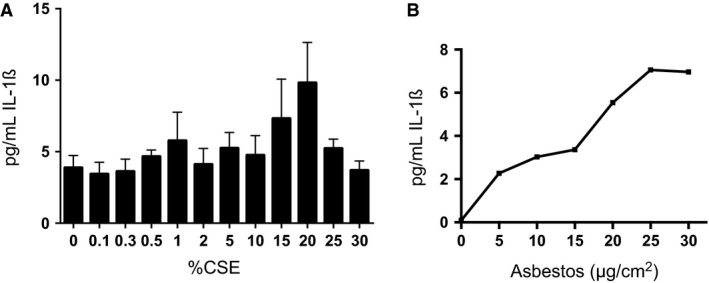
Titration of cigarette smoke extract and asbestos with THP‐1 cells. (A) THP‐1 cells in complete medium were treated with PMA overnight prior to exposure to increasing concentrations of cigarette smoke extract (CSE). After 6 h incubation, levels of IL‐1*β* released into the cell culture supernatant of the CSE‐treated cells were determined in quadruplicate by ELISA. Similar results, that is, limited release of IL‐1*β*, were obtained in a repeat experiment. (B) Same as part A except the next day the cells were treated with increasing amounts of asbestos. After 6 h incubation, levels of IL‐1*β* released into the cell culture supernatant of the asbestos‐treated cells were determined by ELISA. A similar asbestos concentration‐dependent release of IL‐1*β* was observed in an independent experiment.

**Figure 7:**
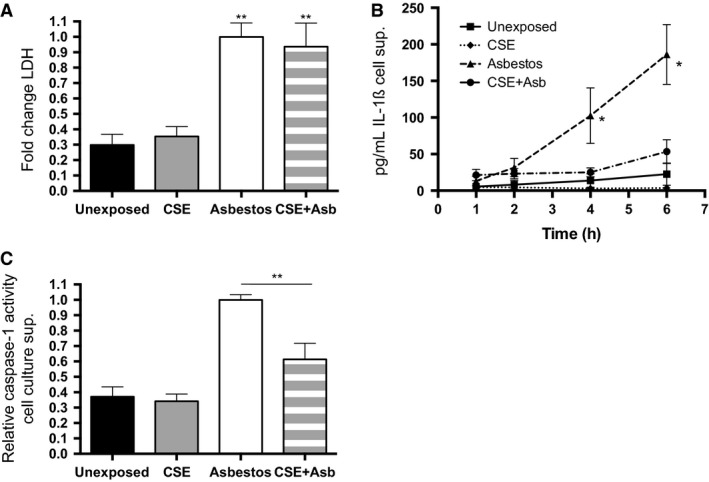
Cigarette smoke extract represses inflammasome activation by asbestos in vitro. THP‐1 cells in complete medium were unexposed or exposed to 20% cigarette smoke extract (CSE) or 10 *μ*g/cm^2^ asbestos or the CSE + asbestos combination. (A) Levels of lactate dehydrogenase (LDH) in the tissue culture media at 6 h postexposure were determined by activity assay. The graph shows the fold change (±SEM) in LDH normalized to the amount in the culture media from asbestos‐exposed THP‐1 cells. The results are from four independent experiments. Asbestos and CSE + asbestos produced significant increases (**P < 0.01) relative to unexposed (*P* = 0.0004, one‐way ANOVA). (B) IL‐1*β* release. Levels of IL‐1*β* in cell culture supernatants of exposed THP‐1 cells were determined by ELISA. Exposure of adherent THP‐1 cells to asbestos induced the time‐dependent release of IL‐1*β* into the culture media (triangles). Addition of CSE repressed the asbestos‐induced release of IL‐1*β* from THP‐1 cells (circles). The graph shows the mean (±SEM) levels of released IL‐1*β* versus time from five independent experiments (*P = 0.016 asbestos vs. unexposed). (C) Extracellular caspase‐1. Levels of active caspase‐1 in the culture media of exposed THP‐1 cells were determined by activity assay. The graph shows the units of caspase‐1 per *μ*L cell culture media for the indicated exposure. The results are from three independent experiments (*P* < 0.0001, one‐way ANOVA, **P < 0.01 asbestos vs. CSE + asbestos).

Alveolar macrophages are central mediators of the lung's response to inhaled asbestos (Morris and Brody 2007). We tested the effects of cigarette smoke extract (CSE) or asbestos exposure on a human monocytic cell line, THP‐1 cells that can be differentiated into macrophage‐like cells with phorbol myristate acetate (PMA). Measuring extracellular IL‐1*β* released from THP‐1 cells can monitor activation of the NLRP3 inflammasome, which appears to be integral to the macrophage response to asbestos (Dostert et al. 2008). In titration experiments, exposure of adherent THP‐1 cells for 6 h to increasing amounts of CSE (Fig. 6A) or asbestos (Fig. 6B) induced release of IL‐1*β* into the cell culture supernatant. Combined exposures were performed at 20% CSE and 10 *μ*g/cm^2^ asbestos. In the combined exposures, CSE had little effect upon cell viability as determined by release of lactate dehydrogenase (LDH), an intracellular enzyme that is released upon cell lysis (Fig. 7A), while exposure to asbestos increased LDH and the combined CSE + asbestos did not induce additional THP‐1 cell lysis. Addition of asbestos at 10 *μ*g/cm^2^ to the adherent cells stimulated the time‐dependent release of IL‐1*β* into the cell culture supernatant (Fig. 7B, triangles). Inclusion of CSE to the asbestos‐containing medium significantly reduced the release of IL‐1*β* (Fig. 7B, circles). Similar findings were observed regarding the asbestos‐induced release of caspase‐1 into the cell culture supernatant, which was also significantly repressed by the addition of CSE (Fig. 7C).

**Figure 8:**
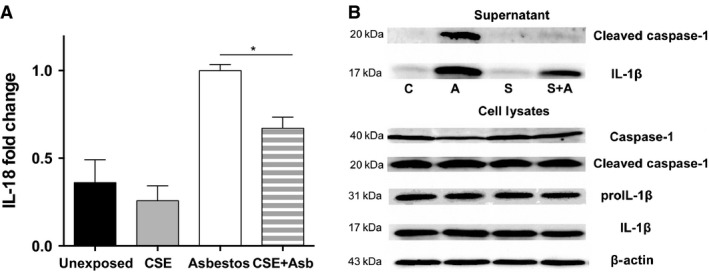
Coexposure to cigarette smoke extract represses release of IL‐18, mature IL‐1*β*, and active caspase‐1 by asbestos‐exposed THP‐1 cells. THP‐1 cells in reduced serum medium (Opti‐mem) were unexposed or exposed to 20% cigarette smoke extract (CSE) or 20 *μ*g/cm^2^ asbestos or the CSE + asbestos combination. (A) After 6 h, IL‐18 levels in the culture media of the cells were determined by ELISA. The graph shows the fold change in released IL‐18 normalized to the amount in the culture media from asbestos‐treated THP‐1 cells. The results are from four independent experiments (*P* < 0.0001 one‐way ANOVA, *P < 0.05 asbestos vs. CSE + asbestos). (B) The cell culture supernatant was concentrated and analyzed for the active (cleaved) form of caspase‐1 and the mature (cleaved) form of IL‐1*β* by immunoblotting. Whole cell extracts were immunoblotted for the pro‐ and active forms of caspase‐1 and IL‐1*β*. A blot for *β* actin was performed to demonstrate equal loading of the cell extract. The lanes are C, control; A, asbestos; S, cigarette smoke extract; S + A, cigarette smoke extract + asbestos. Similar results were obtained in an independent experiment.

To achieve more robust signal for the purpose of assessing NLRP3 inflammasome activation by immunoblotting, we exposed THP‐1 cells to higher levels of asbestos (20 *μ*g/cm^2^) in serum‐free conditions. Under these conditions, no significant increase in cell lysis relative to 10 *μ*g/cm^2^ asbestos, as measured by extracellular LDH, was observed (data not shown). Exposure of THP‐1 cells to 20 *μ*g/cm^2^ asbestos increased levels of IL‐18, another target of the NLRP3 inflammasome, in the cell culture supernatant of THP‐1 cells and addition of CSE repressed its release (Fig. 8A). Immunoblotting of cell lysates from the treated THP‐1 cells revealed little difference in cellular levels of pro‐ or active caspase‐1 or the cleaved or uncleaved forms of IL‐1*β* (Fig. 8B). In contrast, immunblots of concentrated cell culture supernatants confirmed that exposure of THP‐1 cells to 20 *μ*g/cm^2^ asbestos induced release of active cleaved caspase‐1 and mature cleaved IL‐1*β* and that addition of CSE to the asbestos‐containing media repressed that induction. These data demonstrate that CSE reduces inflammation by repressing asbestos‐mediated activation of the NLRP3 inflammasome.

## Discussion

We show here using subchronic exposure that CS represses activation of the innate immune response to inhaled asbestos in a well‐established animal model. Although indicators of injury were comparable in asbestos‐ and CS + asbestos‐exposed mice, well‐characterized mediators of innate immunity (caspase‐1, IL‐18, and IL‐33) were significantly reduced in CS + asbestos‐exposed mice relative to their asbestos‐exposed counterparts. Moreover, CS‐mediated repression of two additional mediators of the innate immune response to asbestos in mice, IL‐1*β* and HMGB1, did not show statistical significance, but displayed similar trends. Even modest CS‐mediated repression of both of these potent mediators likely contributes to changes in the biological response to inhaled asbestos. CS coexposure also repressed the influx of eosinophils associated with asbestos exposure and moderated the size of the typical fibroproliferative lesions that develop in asbestos‐exposed mice. Analogous to our results with mice, CSE + asbestos exposure of macrophage‐like cells in vitro elicited LDH release comparable to that of asbestos alone. However, CSE coexposure repressed NLRP3 inflammasome activation mediated by asbestos exposure in vitro. Similar to the findings here, exposure of mesothelial cells in culture to asbestos elicits release of innate immune mediators and caspase‐1‐dependent cell death or pyroptosis (Bergsbaken et al. 2009; Hillegass et al. 2013; Thompson et al. 2014). Our data show that addition of CSE represses caspase‐1 activation by asbestos without altering the overall extent of cell injury.

Our finding that CS represses the innate immune response to inhaled asbestos adds to a growing body of literature indicating that CS impairs immune surveillance in the lung. CS contains a variety of oxidizing free radicals (Pryor 1997) that injure structural and immune cells of the lung and inactivate intracellular signaling and thereby contributes to immune dysfunction (Feldman and Anderson 2013). CS compromises both the function of the mucociliary escalator and the integrity of epithelial cell tight junctions (Mehta et al. 2008) as well as other protective functions of airway epithelial cells (Feldman and Anderson 2013). In addition, CS represses phagocytosis of pathogens by alveolar macrophages (Drannik et al. 2004; Marti‐Lliteras et al. 2009; Phipps et al. 2010) and the activity of the toll‐like and nucleotide oligomerization domain receptors, TLR3, TLR4, NOD1, and NOD2 in these cells (Droemann et al. 2005; Gaschler et al. 2008; Hristova et al. 2012). CS also represses antiviral immunity by impairing RIG‐I activation (Wu et al. 2014). The data here add to these findings by showing that cigarette smoke represses mediators of innate immunity that respond to inhaled asbestos, including IL‐18 and IL‐33. Since active caspase‐1 promotes secretion of HMGB1, IL‐1*β*, and IL‐18 (Sansonetti et al. 2000; Lamkanfi et al. 2010), our demonstration that cigarette smoke extract represses caspase‐1 activation in vitro correlates with reduced expression of these mediators of innate immunity in mice exposed to CS + asbestos. The focus here is on the NLRP3 inflammasome in immune cells, but particulates also activate the NLRP3 inflammasome in lung epithelial cells, which contribute a less robust response per cell that could become significant as increasing numbers of cells respond to injury in more chronic exposures (Hirota et al. 2012; Peeters et al. 2013). Since our CS exposure system is whole body, we cannot discount the possibility that ingestion of toxic CS components through grooming also contributes to immune dysfunction.

IL‐33 is released in a caspase‐1‐independent manner (Ohno et al. 2009; Talabot‐Ayer et al. 2009). Indeed, active caspase‐1 downmodulates the activity of IL‐33 (Cayrol and Girard 2009; Madouri et al. 2015). The relationship between CS‐mediated repression of both IL‐33 release and the NLRP3 inflammasome remains unclear. Recent findings in a CS + viral infection model demonstrate that CS represses group 2 innate lymphoid cell (ILC2) function and generally suppresses Th2‐like responses (Kearley et al. 2015). CS also increases expression, but not release, of IL‐33 from lung epithelial cells, which leads to elevated release of IL‐33 upon subsequent viral infection. In the context of ILC2 repression by CS, virus‐mediated release of IL‐33 leads to skewing of the immune phenotype toward a Th1‐like response (Kearley et al. 2015). The CS + viral infection model can be used to interpret some of the findings here. Consistent with CS‐mediated Th2 repression, our data show that CS reduces lung eosinophilia and fibrogenic lesions induced by inhaled asbestos. In contrast to the CS + viral infection model, our data show that inhaled asbestos does not promote enhanced IL‐33 release upon coexposure with CS; instead, CS represses asbestos‐mediated induction of IL‐33 release. Conceivably, inflammasome activation potentiates asbestos‐mediated release of IL‐33 from the injured epithelium. Such a scenario would connect CS‐mediated repression of the NLRP3 inflammasome and reduced release of IL‐33 and potentially account for the previous ambiguity regarding IL‐33 processing and the NLRP3 inflammasome (Li et al. 2008).

Elimination of immune cells by toxic substances in CS could account for its immunosuppressive effects (Aoshiba et al. 2001). However, histopathologic and electron microscopic studies have shown that alveolar macrophages from smokers are dramatically increased in number and contain multiple inorganic inclusions (Brody and Craighead 1975). These cells also have an increased life span (Marques et al. 1997). In our experiments, CS exposure has little effect on a marker of cell injury, extracellular LDH, in vivo or in vitro with or without asbestos coexposure. Thus, a mechanism that accounts for CS‐mediated repression of the innate immune response to inhaled asbestos remains undetermined, but based on our studies presented here is likely related to repression of inflammasome activation. If CS leads to inflammasome repression via multiple mechanisms, identifying individual contributions will likely be challenging.

Our data show that CS represses several of the well‐characterized components of the innate immune response to inhaled asbestos. We postulate that this immunosuppressive effect would delay fiber clearance and thereby prolong the inflammatory response to inhaled asbestos. Consistent with this view, CS promotes retention of asbestos in the lungs of humans and animal models (McFadden et al. 1986; Churg et al. 1987; Churg and Stevens 1995). Moreover, the reduced clearance of fibers and the corresponding failure to resolve fibrogenesis would account for previous findings that fibroproliferative lesions are worse in rodents instilled with asbestos and exposed to CS for 6 months relative to their counterparts exposed only to asbestos (Tron et al. 1987). We did not observe this enhanced effect in our studies, and the reason could simply be because our experiments did not allow sufficient time for the asbestos lesions to resolve. After a single 5 h exposure, the asbestos lesions show little evidence of resolution even at 30 days postexposure (Chang et al. 1988). Consequently, the experiments described here were designed to detect lesion development instead of lesion resolution. A longer period of observation postexposure would be required to test a postulate considering the smoke‐mediated delay of both fiber clearance and lesion resolution.

According to our hypothesis, the data in this article show that components of the innate immune response that protect the lungs from toxic agents such as asbestos are compromised by cigarette smoke. How the fibroproliferative and neoplastic sequelae will proceed in long‐term coexposures to these agents have yet to be established in the animal model. We have chosen to use chrysotile asbestos in these assays since chrysotile constitutes 95% of the world's use of asbestos (Case et al. 2011) and clearly causes all of the types of asbestos‐induced diseases (Henderson et al. 2004; Markowitz 2015). Also, chrysotile – the asbestos variety used throughout most of our prior published work (e.g., Brody and Roe 1983, Chang et al. 1988, McGavran and Brody 1989, McGavran et al. 1990, Pinkerton et al. 1983), while not as potent as the amphibole asbestos varieties in causing mesothelioma (Henderson et al. 2004; Markowitz 2015) – appears comparable to amphiboles in causing lung cancer (Berman and Crump 2008). Although the NLRP3 inflammasome appears not to be involved in development of mesothelioma (Chow et al. 2012), its role in lung tumorigenesis remains to be evaluated. If activation of the NLRP3 inflammasome facilitates fiber removal from the lung, one might predict that NLRP3 null mice would show enhanced susceptibility to lung tumorigenesis induced by asbestos. However, it is important to consider that partial repression of the NLRP3 inflammasome by CS, as shown here, may lead to chronic low‐level activation, which would not be replicated in NLRP3 null mice. Inflammation can limit tumor growth, but chronic inflammation may lead to induction of immune regulatory responses that can promote tumorigenesis. Therefore, delayed clearance of asbestos as reported previously (McFadden et al. 1986; Churg et al. 1987; Churg and Stevens 1995), and the consequent chronic inflammation, but repressed innate immune response shown here, may account for the enhanced rate of lung cancer in individuals exposed to both CS and asbestos (Henderson et al. 2004; Markowitz et al. 2013; Markowitz 2015).

## Conflict of Interest

None declared.
